# Stability of fatty acid composition of intramuscular fat from pasture- and grain-fed young bulls during the first 7 d postmortem

**DOI:** 10.5194/aab-63-45-2020

**Published:** 2020-02-12

**Authors:** Alberto Horcada, Oliva Polvillo, Pedro González-Redondo, Adoración López, David Tejerina, Susana García-Torres

**Affiliations:** 1 Departamento de Ciencias Agroforestales, Escuela Técnica Superior de Ingeniería Agronómica, Universidad de Sevilla, 41013 Sevilla, Spain; 2 Servicio General de Investigación Agraria (CITIUS), Universidad de Sevilla, 41013 Sevilla, Spain; 3 Instituto de Investigaciones Agrarias Finca La Orden – Valdesequera, Junta de Extremadura, 06187 Guadajira, Spain

## Abstract

In order to study the effect of different amounts of concentrate feed and the effectiveness of natural antioxidants on the fatty acid stability of intramuscular fat during the first days postmortem, 75 young bulls of the Retinta breed were divided in three groups: 30 were grazed, 30 were fed on medium concentrate diets, and 15 were fed on high-concentrate diets. Young bulls were slaughtered at commercial weight, around a 500 kg final body weight. Samples from *Longissimus lumborum* muscle were assigned to two ageing periods (0 and 7 d) and were vacuum packaged in vacuum bags ($O_{2}$ permeability: 9.3 mL $O_{2}$/$m^{2}$ per 24 h at 0 ${}^{\circ }C$) using an EGARVAC${}^{\text{®}}$ sealer. Beef from grass-fed bulls showed a higher polyunsaturated fatty acid content than concentrate-fed bulls. During the first 7 d postmortem, no changes in the fatty acids profile were observed, because α-tocopherol content was optimal to prevent lipid oxidation. The higher level of natural antioxidants in grass than in grain resulted in the stability of the fatty acid profile. This study shows that the anti-oxidative potential of natural antioxidants in meat plays an important role during the first 7 d postmortem.

## 1 Introduction

Meat is considered to be a nutritive food for human consumption because it includes a high content of lipids, proteins, vitamins, and minerals. It is widely reported that beef fat includes polyunsaturated essential fatty acids (n-3 and n-6) that are known to have positive effects on human health (Alfaia et al., 2009). In fact, the intake of n-3 fatty acids in human nutrition is recommended because it leads to multiple health benefits.

During the ageing of fresh meat, a natural autoxidation of lipids occurs (Mungure et al., 2016) and most precursors responsible for flavour and taste are produced. However, an excessive increase in lipid oxidation, associated with long periods of ageing can lead to a decrease in nutritional value and even its acceptability (Scollan et al., 2006). For example, lipid oxidation in meat produces malondialdehyde (MDA) and other oxidized products which are toxic for consumers and need to be controlled, even below the acceptance threshold for rancid off-flavour meat, which is evaluated at 1 µg MDA g${}^{-1}$ of meat (Campo et al., 2006).

In the response to oxidation in intramuscular fat, three main factors should be considered, namely the polyunsaturated fatty acid (PUFA) content, the amount of reactive oxygen species produced, and the level of natural antioxidants in the meat (Daley et al., 2010). PUFAs show a high susceptibility to oxidation because they are known to act as substrates which initiate the oxidative process in meat. Hydroperoxides are the primary products of lipid oxidation, but hydroperoxides, despite their deleterious effects on health, have no effect on the flavour quality of foods. However, these unstable molecules decompose readily to form a myriad of products such as aldehydes, ketones, alcohols, and hydrocarbons, amongst others. To avoid the oxidation of meat, natural antioxidant agents are needed. Antioxidants are chemical compounds that are capable of donating hydrogen to the free radicals in fatty acids to retard lipid peroxidation and reduce damage to the sensory or nutritional properties of meat (Lahucky et al., 2010). The diet of animals contains native antioxidants which counteract the action of pro-oxidants in meat (De la Fuente et al., 2009). Natural antioxidants of lipids are found in almost all plants and even in animal tissues. These include carotenoids, flavonoids, and other plant pigments found in pasture or grass diets. In fact, Jung et al. (2010) reported that feeding animals with forage or grass containing antioxidant compounds could serve to retard the lipid peroxidation of meat.

The oxidative mechanisms of lipids in beef have been reported during long ageing periods (Franco et al., 2012), but there have been no studies of the stability of fatty acid profiles in the first postmortem period. The beef industry usually recommends prolonged periods of maturation for the meat of young bulls to improve its characteristics of texture, colour, and aroma (e.g. 14–21 d of ageing). However, in the Spanish market, the carcasses of young bulls tend to leave the slaughterhouse to be distributed to the different outlets less than 7 d postmortem (Ruiz de Huidobro et al., 2003) in order to avoid weight loss during refrigeration and preserve the red meat colour that pleases the consumer.

Beef production in southwest Spain is mainly based on pure breeds as Retinta, raised under a semi-extensive system in the Dehesa. Retinta is a local Spanish beef breed of early maturity and large size when fully grown (Piedrafita et al., 2003). Calves are reared with their dams until weaning at about 7–8 months of age and before becoming young bulls, and they are transferred to commercial feedlots where they are usually fed *ad libitum* high-concentrate diets, comprised of cereal–soybean meal-based concentrates and cereal straw, given separately during the fattening period in order to promote maximum daily gain and adequate rumination.

Most studies have used beef steaks to investigate the influence of including exogenous antioxidants in the diet on the palatability of beef during long ageing to reduce its susceptibility to fatty acid oxidation. However, few studies have been conducted to explore the interaction between natural sources of antioxidants in the diet of young bulls and the stability of lipids during the early postmortem period in meat (Pouzo et al., 2016). Therefore, the aim of this work was to study the influence of a production system based on the use of different levels of concentrate feed in the diet and the effectiveness of natural antioxidants on the stability of fatty acids (particularly with regard to long-chain fatty acids) of intramuscular fat during the first 7 d postmortem in young bulls of the Retinta breed.

## 2 Material and methods

### 2.1 Animals and diets

**Table 1:** Chemical composition of concentrates used in the treatments, based on grass pasture (GP) and medium (MC) and high (HC) levels of concentrate in the diet.

Composition (dry matter basis)	GP${}^{a}$	MC${}^{a}$	HC${}^{b}$
Raw protein (percent)	12.1	10.4	13.7
Raw fat (percent)	2.5	5.4	6.0
Ash (percent)	6.9	5.9	6.2
Neutral detergent fibre (percent)	62.4	32.8	23.6
Metabolizable energy (megajoules per kilogram)	8.3	11.8	12.8

${}^{a}$ Ingredients (percent of feed): barley grain (36.2); oat grain (24.5); peas (16.6); sunflower seed cake (19.6); and minerals and vitamins (3.1).${}^{b}$ Ingredients (percent of feed): maize grain (34.0); barley grain (33.5); corn gluten feed (17.1); soybean meal 44 (8.4); minerals and vitamins (3.9); and palm oil (3.1).

**Table 2:** Fatty acid profile (expressed as percent of total fatty acids detected) in the treatments based on grass pasture (GP) and medium (MC) and high (HC) levels of concentrate in the diet.

	GP		MC		HC	
	Concentrate	Pasture	Concentrate	Forage${}^{a}$	Concentrate	Forage${}^{b}$
14:0	1.73	2.80	1.88	1.08	2.05	1.37
16:0	21.71	18.42	22.29	20.75	24.90	22.78
16:1	0.84	1.80	0.76	0.42	1.14	0.89
18:0	2.35	3.04	2.92	4.30	3.50	3.99
18:1	36.76	14.83	33.81	20.28	31.86	19.54
18:2	31.32	19.77	30.24	16.06	27.39	16.65
18:3	2.29	34.93	3.58	30.86	2.42	29.14
∑n-6	31.82	20.20	30.97	16.62	28.33	17.29
∑n-3	2.79	35.36	4.31	31.42	3.36	29.78
Others	2.50	4.00	3.80	5.69	5.80	5.01

${}^{a}$ Composed of barley straw and grass silage. ${}^{b}$ Composed of barley straw.

The experiment was carried out using 75 Retinta bulls on three experimental farms in southwestern Spain. The young bulls were reared with their mothers until weaned at 8–9 months on local farms from the National Association of Selected Cattle Breeders of the Retinta breed using mother's milk and grass. At weaning, the young bulls were transferred to three experimental groups ensuring that the mean weight of each group was comparable, as follows. The first group (grass pasture, GP; n=30) was kept at the Research and Technological Development Service of the Government of Extremadura (Badajoz, Spain). The young bulls were fed a diet based on natural grass pasture resources from an agro-silvo-pastoral system called Dehesa, and they also received concentrate in controlled feeders when grass pasture was short (approximately 20 % of the total dry matter supplied). The animals had free access to water in natural resources and waterers. The second group (medium concentrate, MC; n=30) was housed in two pens at the Divino Salvador Cooperative (Cádiz, Spain), allowing 8 $m^{2}$ per animal. The young bulls were fed in controlled feeders with 40 % concentrate, 25 % barley straw, and 35 % grass silage (dry matter basis). The third group (high concentrate, HC; n=15) was allotted to the Diputación de Cádiz Research Farm Station (Cádiz, Spain). The young bulls were permanently housed in pens allowing 4 $m^{2}$ per animal and were fed on concentrate *ad libitum* (approximately 80 % of total dry matter supplied) and barley straw until slaughtered. All concentrate feed included cereals and oilseed grains (Table 1). The animals were weighed on the experimental farms at the beginning and end of the fattening period. Samples of concentrate were collected once a month and analysed to determine dry matter, crude protein, crude fat, and ash according to the AOAC (1990). The concentration of neutral detergent fibre was analysed, and metabolizable energy was calculated according to the NRC (2001). Two samples of pasture, forage, and concentrates were collected at the last month of the fattening period to determine the fatty acid profile of feeds following the method proposed by Juárez et al. (2008). The fatty acid profiles of feeds used from all the experimental groups are shown in Table 2.

All these procedures were conducted according to the guidelines of EU Directive 2010/63/EU on the protection of animals used for experimental and other scientific purposes.

### 2.2 Slaughter procedure and sampling

When young bulls are slaughtered for the local market in Spain, the desired final body weight is around 500 kg (Mercamadrid, 2018), and these animals were slaughtered at an average weight of 517±49 kg. Young bulls were slaughtered in 2 years (15 young bulls each year from each group) at their commercial weight in the local market in licensed slaughterhouses, following the guidelines set out in Council Regulation (EC) No. 1099/2009 (European Communities, 2009) on the protection of animals at the time of killing. Fifteen young bulls from the same group were slaughtered the same day. Effect year was included as a random factor in the statistical model.

At 24 h postmortem, the *Longissimus lumborum* (LL) muscle from the left half of the carcasses was extracted. A steak of 2 cm thickness from cranial portion of LL muscle was excised, which was immediately vacuum packed ($T_{0}$) and stored at -20 ${}^{\circ }C$. Next portion of the LL muscle was preserved for 6 d in a transparent oxygen-permeable polyvinyl chloride film at 4 ${}^{\circ }C$ in a refrigerator (Infrico AN 1002). Then, a steak of 2 cm of thickness from the LL muscle was obtained, vacuum packed, identified ($T_{7}$), and stored at -20 ${}^{\circ }C$. $T_{0}$ and $T_{7}$ were used to determine the fatty acid profile, lipid oxidation analysis, and natural antioxidants (α-tocopherol).

### 2.3 Determination of fatty acid composition

Fatty acid methyl esters (FAMEs) from the intramuscular fat deposit from $T_{0}$ and $T_{7}$ were obtained using the method proposed by Aldai et al. (2006). Separation of FAMEs was carried out using an Agilent 6890N Network gas chromatograph system (Agilent, Inc., Palo Alto, California, USA), equipped with a flame ionization detector (FID) and fitted with a HP-88 capillary column (100m×0.25mm i.d.; 0.2 µm film thickness; Agilent Technologies Spain S.L., Madrid, Spain). The specific conditions of gas chromatography analysis have been reported in Horcada et al. (2016). Individual FAMEs were identified by comparing their retention times with those of authenticated commercial standards from Sigma (Sigma Chemical Co. Ltd., Poole, UK). Individual fatty acids and groups of saturated (SFA), monounsaturated (MUFA) fatty acid, and PUFA were expressed as a percentage of total fatty acids identified.

### 2.4 Lipid oxidation analysis

To analyse lipid stability, the content of thiobarbituric acid reactive substances (TBARs) was determined, according to the method proposed by Salih et al. (1987) with minor modifications. $T_{0}$ and $T_{7}$ samples (2.5 g) were homogenized for 1 min with an ULTRA-TURRAX T 25 digital homogenizer (IKA-Werke GmbH & Co. KG, Staufen, Germany) at 16 000 rpm with 7.5 mL perchloric acid (3.86 %) and 0.25 mL butylated hydroxytoluene (BHT; 4.2 % in ethanol) solution. After homogenization, the samples were filtered through a fast-flow filter paper (90 mm diameter and 100 pk${}^{-1}$) and centrifuged at 2000 rpm for 2 min. The supernatant was collected in a 10 mL erlenmeyer and diluted with 3.86 % perchloric acid, and a 2 mL aliquot of the supernatant was mixed with 2 mL of thiobarbituric acid (TBA) (0.02 M) in distilled water and stored in capped tubes. The tubes were kept in a water bath heated at 90 ${}^{\circ }C$ for 30 min and cooled under tap water for 10 min. Absorbance was measured at 532 nm with a spectrophotometer (Cary 60 UV-Vis-NIR, Agilent). TBARs values were expressed as milligrams of MDA per kilogram of fresh meat using a standard curve prepared from a solution of 1,1,3,3-tetraethoxypropane in the $1\times 10^{-8}$ to $8\times 10^{-8}$ M range.

### 2.5 α-tocopherol analysis

Duplicate samples were made, and the α-tocopherol levels in LL muscle were determined at $T_{0}$ and $T_{7}$ using high-performance liquid chromatography (HPLC), as described by Cayuela et al. (2003). Tocopherol extract was obtained by homogenizing a 1 g sample of muscle, 250 mg of ascorbic acid, 7.5 mL of saponification solution (11.5 % KOH in ethanol/$H_{2}O$; 55:45), and 4 mL of 0.01 % BHT in isooctane. The samples were heated at 80 ${}^{\circ }C$ for 15 min. After cooling, the samples were centrifuged at 1500 rpm for 5 min, and the upper layer was collected for HPLC analysis. α-tocopherol determination was performed using an Agilent Technologies HPLC Series 1100 instrument (Agilent Technologies, Santa Clara, CA, USA) equipped with a Kromasil silica 150cm×4.6 cm (5 µm) column (Kromasil 100-5-SIL, Symta, Madrid, Spain) and a Kromasil silica guard column (10 µm) (Kromasil 100-10-C4, Symta, Madrid, Spain). The thermobile phase was isooctane/tetrahydrofurane (97:3) at a flow rate of 1 $\mathrm{mL}\;\min^{-1}$. The HPLC system was equipped with a G1311A quaternary pump (Agilent Technologies, Santa Clara, CA, USA) and a G1379A degasser (Agilent Technologies, Santa Clara, CA, USA). The fluorescence detector (Agilent Technologies; 1200 Series FLD G1321A) was fixed at λ-excitation = 295 and λ-emission =330 nm. Identification and quantification of the peaks were carried out by comparison with α-tocopherol and standards from Sigma Aldrich in the 0.2–14 $µg\;{\mathrm{mL}}^{-1}$ range (Sigma-Aldrich, St Louis, USA). The results were expressed as micrograms of α-tocopherol per gram of muscle.

### 2.6 Statistical analysis

The general linear model (GLM) was used to analyse the influence of the three different feeding regimes, the two postmortem periods, and their interaction on the fatty acid profiles of intramuscular fat, TBA results, and α-tocopherol content in beef. Previous statistical analysis showed that no year effect existed in this trial. The effect of carcass weight was used as a covariate. Post-hoc multiple comparisons were made using Bonferroni tests when the feeding regime was significant (P<0.05). The statistical models that were used included the fixed effects of feeding regime and postmortem period, as well as the individual as a random effect. The model used was as follows:

1 $$Y_{ijk}=\mu +{\text{FR}}_{i}+{\text{PT}}_{j}+{\text{FR}}_{i}\times {\text{PT}}_{j}+e_{ijk},$$

where $Y_{ijk}$ is the individual or group of fatty acid (expressed as a percent of the total fatty acids identified), MDA (in milligrams of MDA per kilogram of meat), or α-tocopherol (in micrograms per kilogram of meat) content; μ is the least squares mean value; FR${}_{i}$ is the fixed effect of feeding regimes (i=1: grass pasture; i=2: medium concentrate; and i=3: high concentrate); PT${}_{j}$is the fixed effect of postmortem time (j=1: first day; j=2: seventh day); ${\text{FR}}_{i}\times {\text{PT}}_{j}$ is the interaction between the feeding regimes and postmortem time; $e_{ijk}$ is the random residual. Differences were considered statistically significant when P<0.05. The SPSS v.15.0 (SPSS Inc., Chicago, IL, USA) program was used to perform all the statistical analyses.

## 3 Results and discussion

### 3.1 Fatty acid composition of meat

Fatty acid composition of intramuscular fat expressed as a percentage of the total fatty acids detected is shown in Table 3. The highest percentage of SFA in beef was observed in animals from two concentrate-fed groups (48.0 % and 49.0 % for MC and HC, respectively). The highest percentage of MUFA content was observed in grain-fed beef (39.4 % and 38.8 % for MC and HC, respectively), while pasture-fed beef showed the highest percentage of PUFA (18.7 %) among all production systems proposed. The amount of SFA and MUFA increased in meat from concentrate-fed animals when compared to animals fed on pasture diets, which is mainly due to an increase in 16:0 and 18:1 fatty acids in grain-fed young bulls.

**Table 3:** Effect of feeding regimes (grass pasture, GP; medium level concentrate, MC, and high level concentrate, HC) and postmortem time ($T_{0}$ and $T_{7}$ represent 0 and 7 d, respectively) on fatty acid profile (expressed as a percent of the total fatty acids detected) in intramuscular fat from young Retinta bulls.

	Feeding regime				Postmortem time			SEM		Effects		
	GP	MC	HC		$T_{0}$	$T_{7}$				FR	PT	FR × PT
SFA	47.10${}^{b}$	48.02${}^{a}$	49.18${}^{a}$		47.79	48.36		0.301		<0.001	0.061	<0.001
MUFA	34.18${}^{b}$	39.41${}^{a}$	38.85${}^{a}$		36.84	36.87		0.288		<0.001	0.087	<0.001
PUFA	18.71${}^{a}$	12.57${}^{b}$	11.97${}^{b}$		15.37	14.77		0.151		<0.001	0.070	<0.001
8:0	0.05${}^{a}$	0.04${}^{b}$	0.04${}^{b}$		0.05	0.04		0.001		<0.001	0.069	0.025
10:0	0.08${}^{b}$	0.15${}^{a}$	0.07${}^{b}$		0.12	0.10		0.006		<0.001	0.290	0.791
11:0	0.03${}^{a}$	0.03${}^{a}$	0.02${}^{b}$		0.03	0.03		0.001		0.009	0.145	0.906
12:0	0.14${}^{b}$	0.25${}^{a}$	0.28${}^{a}$		0.23	0.19		0.011		<0.001	0.139	0.763
13:0	0.02${}^{b}$	0.03${}^{a}$	0.03${}^{a}$		0.03	0.03		0.001		<0.001	0.787	0.959
14:0	2.2${}^{b}$	2.7${}^{a}$	2.9${}^{a}$		2.5	2.6		0.034		<0.001	0.212	0.383
14:1	0.29${}^{b}$	0.38${}^{a}$	0.35${}^{a}$		0.33	0.35		0.008		<0.001	0.558	0.583
15:0	0.38${}^{b}$	0.51${}^{a}$	0.39${}^{b}$		0.43	0.44		0.007		<0.001	0.311	0.297
15:1	0.08${}^{b}$	0.11${}^{a}$	0.08${}^{b}$		0.10	0.09		0.005		0.017	0.425	0.981
16:0	23.5${}^{b}$	25.2${}^{a}$	24.8${}^{a}$		24.3	24.5		0.087		<0.001	0.090	0.454
16:1	2.3${}^{c}$	3.4${}^{a}$	2.9${}^{b}$		2.8	2.9		0.050		<0.001	0.367	0.259
17:0	0.98${}^{b}$	1.29${}^{a}$	0.99${}^{b}$		1.08	1.13		0.018		<0.001	0.384	0.079
17:1	0.66${}^{b}$	1.12${}^{a}$	0.63${}^{b}$		0.82	0.85		0.024		<0.001	0.705	0.095
18:0	18.5${}^{a}$	16.8${}^{b}$	18.8${}^{a}$		17.8	18.0		0.107		<0.001	0.154	0.651
9t18:1	1.1${}^{a}$	0.5${}^{c}$	0.8${}^{b}$		0.8	0.8		0.033		<0.001	0.788	0.756
11t18:1	0.84${}^{b}$	0.93${}^{b}$	1.37${}^{a}$		0.98	0.98		0.025		<0.001	0.817	0.274
9c18:1	27.7${}^{b}$	32.8${}^{a}$	32.1${}^{a}$		30.6	30.5		0.210		<0.001	0.497	0.195
6t18:2	0.25${}^{b}$	0.26${}^{a,b}$	0.29${}^{a}$		0.26	0.27		0.005		0.012	0.488	0.477
6c18:2	11.4${}^{a}$	7.7${}^{c}$	8.7${}^{b}$		9.6	9.4		0.150		<0.001	0.103	0.656
18:3 (n-6)	0.14${}^{a}$	0.11${}^{b}$	0.07${}^{c}$		0.12	0.10		0.003		<0.001	0.037	0.142
18:3 (n-3)	0.79${}^{a}$	0.50${}^{b}$	0.41${}^{c}$		0.60	0.58		0.015		<0.001	0.025	0.617
20:0	0.26${}^{a}$	0.20${}^{b}$	0.10${}^{c}$		0.20	0.21		0.006		<0.001	0.464	0.194
9c, 11t CLA	0.46${}^{a}$	0.28${}^{c}$	0.36${}^{b}$		0.39	0.39		0.008		<0.001	0.880	0.524
20:1 (n-9)	0.21${}^{b}$	0.20${}^{b}$	0.30${}^{a}$		0.22	0.22		0.005		<0.001	0.595	0.351
21:0	0.07${}^{a}$	0.05${}^{b}$	0.09${}^{a}$		0.07	0.06		0.003		<0.001	0.761	0.943
20:2	0.14${}^{a}$	0.11${}^{b}$	0.10${}^{b}$		0.15	0.14		0.004		<0.001	0.075	0.065
22:0	0.89${}^{a}$	0.53${}^{b}$	0.34${}^{c}$		0.66	0.63		0.025		<0.001	0.548	0.609
20:3 (n-6)	0.05	0.04	0.04		0.05	0.04		0.002		0.608	0.305	0.732
22:1 (n-9)	0.08	0.08	0.06		0.08	0.07		0.004		0.139	0.509	0.642
20:4 (n-6)	3.9${}^{a}$	2.1${}^{b}$	1.7${}^{c}$		2.9	2.7		0.083		<0.001	0.081	0.005
20:3 (n-3)	0.07${}^{a}$	0.05${}^{b}$	0.05${}^{b}$		0.06	0.06		0.002		<0.001	0.208	0.212
23:0	0.06${}^{a}$	0.04${}^{b}$	0.04${}^{b}$		0.05	0.05		0.003		<0.001	0.446	0.158
20:5 (n-3)	0.63${}^{a}$	0.30${}^{b}$	0.09${}^{c}$		0.42	0.37		0.021		<0.001	0.104	0.204
22:2	0.05	0.05	0.04		0.05	0.04		0.002		0.381	0.283	0.531
24:0	0.14${}^{\text{a,b}}$	0.13${}^{b}$	0.19${}^{a}$		0.14	0.15		0.008		0.029	0.512	0.638
24:1	0.07	0.07	0.06		0.07	0.07		0.002		0.092	0.689	0.970
22:5 (n-3)	1.1${}^{a}$	0.6${}^{b}$	0.2${}^{c}$		0.8	0.7		0.029		<0.001	0.160	0.161
22:6 (n-3)	0.18	0.11	0.07		0.13	0.13		0.005		<0.001	0.354	0.192

SFA: saturated fatty acids; MUFA: unsaturated fatty acids; PUFA: polyunsaturated fatty acids; CLA: conjugated linoleic acids; FR: feeding regime; PT: postmortem time; FR × PT: interaction between FR and PT; SEM: standard error of the mean. Values with the same letters (a, b, c) indicate homogeneous subsets for P≤0.05 according to Bonferroni's test.

**Table 4:** Effect of feeding regimes (grass pasture, GP; medium level concentrate, MC, and high concentrate levels, HC) and postmortem time ($T_{0}$ and $T_{7}$ represent 0 and 7 d, respectively) on lipid stability (expressed as TBARs) and α-tocopherol content in meat from young Retinta bulls.

	GP		P value	MC		P value	HC		P value
	$T_{0}$	$T_{7}$		$T_{0}$	$T_{7}$		$T_{0}$	$T_{7}$	
TBARs (mg MDA kg${}^{-1}$ meat)	0.012	0.021	0.220	0.065	0.111	0.031	0.082	0.245	0.037
α-tocopherol (µg g${}^{-1}$ meat)	1.67	1.52	0.469	0.83	0.71	0.147	0.82	0.82	0.980

TBARs: thiobarbituric acid reactive substances; MDA: malondialdehyde; FR: feeding regime; PT: postmortem time; FR × PT: interaction between FR and PT; SEM: standard error of the mean.

Dietary lipids of animals is the most important factor affecting the fatty acid composition of meat (Wood et al., 2008). Influence of the feeding regime on the meat fatty acid content was observed in 35 of 38 fatty acids detected (Table 3). In reference to the quantitatively larger fatty acids, the percentage of oleic (9c18:1) and palmitic (16:0) fatty acids were higher in meat from grain-fed than pasture-fed bulls. Although concentrate feed from the GP system in this study showed the highest percentage of 18:1 fatty acid, meat from the GP group contained significantly lower proportions of 9c18:1 fatty acid than the MC and HC groups (P<0.05). The low accumulation of 18:1 fatty acid in the meat of GP young bulls could be because when the animals are fed based on grass, food stays longer in the rumen than when fed with concentrate, and thus a greater biohydrogenation of 18:1 to 18:0 fatty acid can occur. In these conditions, a higher volatile fatty acid concentration in the rumen (i.e. acetate) is produced from 18:1 fatty acid previous to the synthesis of SFA. On the other hand, Smith et al. (2009) reported that a higher metabolic availability of glucose when the animals are fed concentrate rations increases the expression of lipogenic enzymes, such as stearoyl-CoA desaturase, favouring the accumulation of 9c18:1 fatty acid in fat tissue.

Higher percentages of 18:3 (n-6) and 18:3 (n-3) were observed in meat from pasture-fed young bulls compared to grain-fed young bulls (Table 3). The type and quality of pasture and forage are assumed to affect the proportion of 18:3 fatty acid in meat because linolenic acid (18:3 (n-3)) has been shown to be a major constituent of the total lipid content of grass. As the pasture grass in this study had a higher 18:3 fatty acid content than silage and straw in the MC and HC groups (Table 2), the GP meat samples showed a higher 18:3 fatty acid percentage than the MC and HC samples. As reported by Wood and Enser (1997), the lipid is enclosed by the intact organelle (chloroplast), which provides natural protection for n-3 PUFA by biohydrogenation in the rumen. Thus, these fatty acids have the natural possibility to pass through the rumen and avoid its microbial biohydrogenation activity.

With reference to long-chain fatty acids, a higher percentage of 20:4 (n-6), 20:3 (n-3), 20:5 (n-3), and 22:5 (n-3) fatty acids was observed in meat from pasture-fed than grain-fed young bulls. These results are in agreement with Yang et al. (2002), which reported a higher percentage of 20:4 (n-6) and 22:5 (n-3) fatty acids in meat from steers grazed in pasture. The general recommendation for fat intake is to reduce saturated fats and to increase long-chain PUFA n-3 consumption, especially 18:3 (n-3), 20:5 (n-3), 22:5 (n-3), and 22:6 (n-3) fatty acids, because they play an important role in the prevention of heart diseases and some cancers. The sum of n-3 PUFA increased with the reduction of grain in the diet. In fact, meat from pasture-fed young bulls showed twice the amount of the desirable n-3 PUFA than that from grain-fed bulls.

### 3.2 Fatty acids stability

Significant changes in the percentage of fatty acids during the first 7 d postmortem were produced only for the 18:3(n-6) and 18:3 (n-3) fatty acids (Table 3), where a significant decrease in 18:3(n-6) (P<0.05) and 18:3(n-3) (P<0.05) fatty acid content during the first seven postmortem days was observed. These findings were consistent with the results of Wichtel et al. (1996), which reported that serum from dairy heifers grazed on pasture had increased peroxidizable PUFA (i.e. 18:3) concentrations. Therefore, it can be considered that the level of natural antioxidants in the meat of pasture-fed young bulls is particularly insufficient to maintain the stability of 18:3 fatty acid during the first 7 d postmortem, because this fatty acid shows a high susceptibility to oxidation. As reported by Arnold et al. (1993), a minimum deposit of 3 µg of α-tocopherol g${}^{-1}$ of fresh muscle is needed in tissues to protect it from beef oxidation. Thus, the α-tocopherol concentrations observed in our study (1.64 µg of α-tocopherol g${}^{-1}$ fresh muscle) for GP beef could be considered insufficient to minimize the lipid oxidation of the high percentage of 18:3 fatty acid present in meat from pasture-fed young bulls.

Meat containing a greater content of unsaturated fatty acids is more prone to lipid oxidation than that which contains more saturated fat (Yang et al., 2002). The level of lipid oxidation was determined from the TBAR values and expressed as milligrams of MDA per kilogram of meat. Lipid oxidation in meat produces MDA and other oxidized products that can be toxic for consumers, and so they need to be controlled. In fact, TBAR values of about 0.5 mg MDA kg${}^{-1}$ meat are considered critical, since at this level of lipid oxidation, a rancid odour and taste can be detected by consumers (Wood et al., 2008). In the present study, the TBAR values in the meat did not exceed this critical level between 0 and 7 d postmortem (Table 4) and were lower than those reported by Campo et al. (2006) in Angus and Charolais cross steers (values above 1 mg MDA kg${}^{-1}$).

Franco et al. (2012) reported that the growth of TBARs in beef was affected by a triple interaction between animal diet, time ageing, and antioxidant content in the meat. Two factors (feeding regime and postmortem time) and their interaction had a significant effect (P<0.001) on the MDA concentration in meat. The mean TBAR concentration for meat from the highest grain feeding regimes (MC and HC) were significantly higher than in samples from GP (P<0.05). On the other hand, the influence of ageing for 7 d postmortem was observed (P<0.001) in the TBAR values. In fact, a significant increase in MDA content in the meat at 7 d post mortem was observed in all groups (Table 4). These results are in agreement with Yang et al. (2002) and Campo et al. (2006), which reported a nonlinear increase in TBAR values in beef during the 7 d ageing period. In fact, this increase in TBAR values mainly occurs from 4 d postmortem in animals because lipid oxidation is a free-radical autocatalytic chain reaction. The TBAR values increased 2-fold in MC, while in the highest grain (HC) diet , they increased dramatically (3-fold) during the first seven postmortem days. No significant changes in TBAR values were observed in the GP group (p>0.05). This observation could related to the higher natural antioxidant content (α-tocopherol) observed in grass-fed meat than grain-fed meat (Table 4). Antioxidant properties of the α-tocopherol on lipids stability have been reported (Wood et al., 2008). Tocopherols are constituents of a series of benzopyranols present in vegetables tissues and oils and are powerful lipid-soluble antioxidants (Lampi, 2011). In the current study, the effect of feeding regime of animals on α-tocopherol content was significant (P<0.001), and the content of this natural antioxidant was 2 times more in grass-fed meat (1.64 µg g${}^{-1}$ meat) than in grain-fed beef (MC and HC) (Table 4). These results are in agreement with Nuernberg et al. (2005) and De la Fuente et al. (2009) which reported a higher concentration of α-tocopherol in the muscle from young bulls raised in pasture than grain-fed systems. Warren et al. (2008) reported that diets based mainly in cereal had low natural antioxidant content, and consequently the meat could be oxidatively unstable.

## 4 Conclusions

Regardless of the grain content in the young bulls rations, during the first 7 d postmortem of young Retinta bulls, changes in the stability of lipids leading to a reduction in the acceptance of beef are not be expected because meat includes a sufficient amount of natural antioxidants that prevent acid oxidation in meat. Then, in this period no effect on the flavour quality of the beef can be expected. However, a high natural antioxidant content is required in beef to preserve the stability of 18:3 fatty acid during ageing. In this period, changes leading to oxidation of meat are more important in grain meat that grass meat because it includes less natural antioxidants. Based on the results on the present study, from a nutritional point of view it could be recommended that humans consume cuts of fresh beef from grass-fed young bulls because of the more highly desirable fatty acid content.

## Data Availability

The data from this study can be accessed from the corresponding author upon a reasonable request.

## References

[bib1.bib1] Aldai N, Osoro K, Barrón LJR, Nájera AI (2006). Gas-liquid chromatographic method for analysing complex mixtures of fatty acids including conjugated linoleic acids (*cis*9*trans*11 and *trans*10*cis*12 isomers) and long-chain (n-3 or n-6) polyunsaturated fatty acids: Application to the intramuscular fat of beef meat. J Chromatogr A.

[bib1.bib2] Alfaia CP, Alves SP, Martins SI, Costa AS, Fontes CM, Lemos JP, Bessa RJ, Prates JA (2009). Effect of the feeding system on intramuscular fatty acids and conjugated linoleic acid isomers of beef cattle, with emphasis on their nutritional value and discriminatory ability. Food Chem.

[bib1.bib3] AOAC (1990). Official Methods of Analysis.

[bib1.bib4] Arnold RN, Arp SC, Scheller KK, Williams SN, Schaefer DM (1993). Tissue equilibration and subcellular-distribution of vitamin-E relative to myoglobin and lipid oxidation in displayed beef. J Anim Sci.

[bib1.bib5] Campo MM, Nute GR, Hughes SI, Enser M, Wood JD, Richardson RI (2006). Flavour perception of oxidation in beef. Meat Sci.

[bib1.bib6] Cayuela JM, Garrido MD, Sancho-Bañón JS, Ros JM (2003). Simultaneous HPLC analysis of α-tocopherol and cholesterol in fresh pig meat. J Agr Food Chem.

[bib1.bib7] Lampi AM (2011). Analysis of tocopherols and tocotrienols by HPLC, Selected Topics in the Analysis of Lipids.

[bib1.bib8] Daley C, Abbott A, Doyle P, Nader G, Larson S (2010). A review of fatty acid profiles and antioxidant content in grass-fed and grain-fed beef. Nutri J.

[bib1.bib9] De la Fuente J, Díaz MT, Álvarez I, Oliver MA, Font I Furnols M, Sañudo C, Campo MM, Montossi F, Nute GR, Cañeque V (2009). Fatty acid and vitamin E composition of intramuscular fat in cattle reared in different production systems. Meat Sci.

[bib1.bib10] Directive 2010/63/EU (2010). Directive 2010/63/EU of the European Parliament and of the Council of 22 September 2010 on the protection of animals used for scientific purposes. Official Journal of the European Union.

[bib1.bib11] European Communities (2009). Council Regulation (EC) No. 1099/2009 of 24 September 2009 on the protection of animals at the time of killing. Official Journal of the European Communities.

[bib1.bib12] Franco D, González L, Bispo E, Latorre A, Moreno T, Sineiro J, Sánchez M, Núñez MJ (2012). Effects of calf diet, antioxidants, packaging type and storage time on beef steak storage. Meat Sci.

[bib1.bib13] Horcada A, Polvillo O, Juárez M, Avilés C, Martínez AL, Peña F (2016). Influence of feeding system (concentrate and total mixed ration) on fatty acid profiles of beef from three lean cattle breeds. J Food Compos Anal.

[bib1.bib14] Juárez M, Polvillo O, Contò M, Ficco A, Ballico S, Failla S (2008). Comparison of four extraction/methylation analytical methods to measure fatty acid composition by gas chromatography in meat. J Chromatogr A.

[bib1.bib15] Jung S, Choe JH, Kim B, Yun H, Kruk ZA, Jo C (2010). Effect of dietary mixture of gallic acid and linoleic acid on antioxidative potential and quality of breast meat from broilers. Meat Sci.

[bib1.bib16] Lahucky R, Nuernberg K, Kovac L, Bucko O, Nuernberg G (2010). Assessment of the antioxidant potential of selected plant extracts – In vitro and in vivo experiments on pork. Meat Sci.

[bib1.bib17] Ferias, Mercados y Mataderos [Internet].

[bib1.bib18] Mungure TE, Bekhit AEA, Birch EJ, Stewart I (2016). Effect of rigor temperature, ageing and display time on the meat quality and lipid oxidative stability of hot boned beef *Semimembranosus* muscle. Meat Sci.

[bib1.bib19] National Research Council (NRC) (2001). Nutrient requirements of dairy cattle.

[bib1.bib20] Nuernberg K, Dannenberger D, Nuernberg G, Ender K, Voigt J, Scollan ND, Wood JD, Nute GR, Richardson RI (2005). Effect of a grass-based and a concentrate feeding system on meat quality characteristics and fatty acid composition of *Longissimus* muscle in different cattle breeds. Livest Prod Sci.

[bib1.bib21] Piedrafita J, Quintanilla R, Sañudo C, Olleta JL, Campo MM, Panea B, Renand G, Turin F, Jabet S, Osoro K, Oliván MC, Noval G, García P, García MD, Oliver MA, Gispert M, Serra X, Espejo M, García S, López M, Izquierdo M (2003). Carcass quality of 10 beef cattle breeds of the Southwest of Europe in their typical production systems. Livest Prod Sci.

[bib1.bib22] Pouzo L, Descalzo A, Zaritzky N, Rossetti L, Pavan E (2016). Antioxidant status, lipid and color stability of aged beef from grazing steers supplemented with corn grain and increasing levels of flaxseed. Meat Sci.

[bib1.bib23] Ruiz de Huidobro F, Miguel E, Onega E, Blázquez B (2003). Changes in meat quality characteristics of bovine meat during the first 6 days post mortem. Meat Sci.

[bib1.bib24] Salih AM, Smith DM, Price JF, Dawson LE (1987). Modified extraction 2-thiobarbituric acid method for measuring lipid oxidation. Poultry Sci.

[bib1.bib25] Scollan N, Hocquette JF, Nuernberg K, Dannenberger D, Richardson I, Moloney A (2006). Innovations in beef production systems that enhance the nutritional and health value of beef lipids and their relationship with meat quality. Meat Sci.

[bib1.bib26] Smith BK, Robinson LE, Nam R, Ma DW (2009). *Trans*-fatty acids and cancer: A mini-review. Brit J Nutr.

[bib1.bib27] Warren HE, Scollan ND, Nute GR, Hughes SI, Wood JD, Richardson RI (2008). Effects of breed and a concentrate or grass silage diet on beef quality in cattle of 3 ages, II: Meat stability and flavor. Meat Sci.

[bib1.bib28] Wichtel JJ, Freeman DA, Craigie AL, Varela-Álvarez H, Williamson NB (1996). Alpha-tocopherol, selenium and polyunsaturated fatty acid concentrations in the serum and feed of spring-calving dairy heifers. New Zeal Vet J.

[bib1.bib29] Wood JD, Enser H (1997). Factors influencing fatty acids in meat and the role of antioxidants in improving meat quality. Brit J Nutr.

[bib1.bib30] Wood JD, Enser M, Fisher AV, Nute GR, Sheard PR, Richardson RI, Hughes SI, Whittington FM (2008). Fat deposition, fatty acid composition and meat quality: A review. Meat Sci.

[bib1.bib31] Yang A, Lanari MC, Brewster M, Tume RK (2002). Lipid stability and meat colour of beef from pasture- and grain-fed cattle with or without vitamin E supplement. Meat Sci.

